# Shared Genetic Factors Involved in Celiac Disease, Type 2 Diabetes and Anorexia Nervosa Suggest Common Molecular Pathways for Chronic Diseases

**DOI:** 10.1371/journal.pone.0159593

**Published:** 2016-08-02

**Authors:** Joanna Mostowy, Caroline Montén, Audur H. Gudjonsdottir, Henrik Arnell, Lars Browaldh, Staffan Nilsson, Daniel Agardh, Åsa Torinsson Naluai

**Affiliations:** 1 Institute of Biomedicine, The Sahlgrenska Academy at the University of Gothenburg, Gothenburg, Sweden; 2 Diabetes and Celiac Disease Unit, Department of Clinical Sciences, Lund University, Malmö, Sweden; 3 Institute of Clinical Sciences, Sahlgrenska Academy at the University of Gothenburg, Gothenburg, Sweden; 4 Department of Pediatric Gastroenterology, Hepatology and Nutrition, Karolinska University Hospital and Division of Pediatrics, CLINTEC, Karolinska Institutet, Stockholm, Sweden; 5 Department of Clinical Science and Education, Karolinska Institutet, Sodersjukhuset, Stockholm, Sweden; 6 Department of Mathematical Sciences, Chalmers University of Technology, Gothenburg, Sweden; Hospital Israelita Albert Einstein, BRAZIL

## Abstract

**Background and Objectives:**

Genome-wide association studies (GWAS) have identified several genetic regions involved in immune-regulatory mechanisms to be associated with celiac disease. Previous GWAS also revealed an over-representation of genes involved in type 2 diabetes and anorexia nervosa associated with celiac disease, suggesting involvement of common metabolic pathways for development of these chronic diseases. The aim of this study was to extend these previous analyses to study the gene expression in the gut from children with active celiac disease.

**Material and Methods:**

Thirty six target genes involved in type 2 diabetes and four genes associated with anorexia nervosa were investigated for gene expression in small intestinal biopsies from 144 children with celiac disease at median (range) age of 7.4 years (1.6–17.8) and from 154 disease controls at a median (range) age 11.4.years (1.4–18.3).

**Results:**

A total of eleven of genes were differently expressed in celiac patients compared with disease controls of which *CD36*, *CD38*, *FOXP1*, *SELL*, *PPARA*, *PPARG*, *AGT* previously associated with type 2 diabetes and *AKAP6*, *NTNG1* with anorexia nervosa remained significant after correction for multiple testing.

**Conclusion:**

Shared genetic factors involved in celiac disease, type 2 diabetes and anorexia nervosa suggest common underlying molecular pathways for these diseases.

## Introduction

The prevalence of autoimmune disease including celiac disease has increased among the population in many high-income countries worldwide [1, 2]. Celiac disease is an autoimmune intestinal disorder triggered by intolerance to gluten in genetic susceptible individuals carrying the HLA-DR3-DQ2 and DR4-DQ8 risk haplotypes [3]. Another feature of celiac disease is the presence of autoantibodies directed against tissue transglutaminase (tTGA), the serological marker for the disease [4, 5] as well as to protein kinase C delta (*PRKCD*) [6]. It is well established that HLA-DQ heterodimers present gluten peptides to CD4^+^ T-cells causing an inflammation of the gut mucosa leading to villous atrophy with malabsorption of vitamins and nutrients as a consequence [5, 7].

Most chronic inflammatory disorders have a multifactorial etiology caused by a cumulative effect of environmental factors triggering the disease in genetically susceptible individuals [8]. To disentangle which of environmental and/or genetic factors that are involved in disease development and to provide a deeper understanding of the pathogenesis, linkage analysis, allele-sharing methods, genetic association studies in human populations, or genetic analysis of large crosses in model organisms have been developed [9]. Genome-wide association studies (GWAS) enable testing of the whole genome in order to identify statistical association between genetic variants and a trait of interest to compare the frequency of genetic variants (alleles) in affected and unaffected individuals [10].

To date, GWAS has identified more than forty genome-wide significant non-HLA risk loci linked to celiac disease [7, 11–15]. Many of these celiac disease associated loci exhibit an overlap with those of other immune-related diseases such as type 1 diabetes (T1D) [13]. In a previous family GWAS on celiac disease, an overlap between genes implicated in type 2 diabetes (T2D) and anorexia nervosa and gene-regions associated with celiac disease was revealed and a new model behind disease was suggested [11]. Although there is an overlap of celiac disease and other autoimmune conditions, few studies have been performed on the possible common aetiology between celiac disease and metabolic conditions such as T2D and anorexia. Due to the large number of associated polymorphisms and possible bias towards common metabolic pathways, the overlap which was identified in the previous GWAS could be just a chance finding. However, there are also data supporting a connection between these metabolic pathways and celiac disease. A population study from India recently demonstrated an increased frequency of tTGA in T2D patients [16]. Furthermore, a change in metabolism may affect the risk for disease. When a gluten-free diet was introduced in patients diagnosed with celiac disease, it reduced the risk of T2D, even when corrected for BMI [17].

Despite the fact that there can be a high variability of expression of genes in different tissues and between individuals making it a challenge to use this type of data for diagnostic purposes, targeting of genes and expression of them in the affected organ may lead to better understanding of how genes are involved in signaling pathways that eventually lead to chronic diseases such as celiac disease and T2D. The aim of this study was therefore to investigate potential common genetic factors contributing to the development of celiac disease, T2D and anorexia nervosa by analyzing gene expression in intestinal biopsies from children with active celiac disease as compared with disease controls.

## Results

A total of 46 target genes involved in T2D or in anorexia nervosa were identified by pathway enrichment analysis in our previous GWAS study[11]. Six of these genes were analyzed for gene expression previously in our material [11, 18] and the remaining forty genes were selected in this study for gene expression analysis. A pilot gene expression experiment was run on all 40 target genes involved in T2D or anorexia nervosa using 51 celiac disease cases and 64 controls. Out of these 40 target genes, 16 genes showed nominally significant differences in gene expression and were picked along with reference genes for the main experiment including 144 cases and 154 controls. The list of all 46 target genes and control genes are presented in S1 Table (including the six identified T2D genes which were analyzed for gene expression previously [11].)

**Fig 1:**
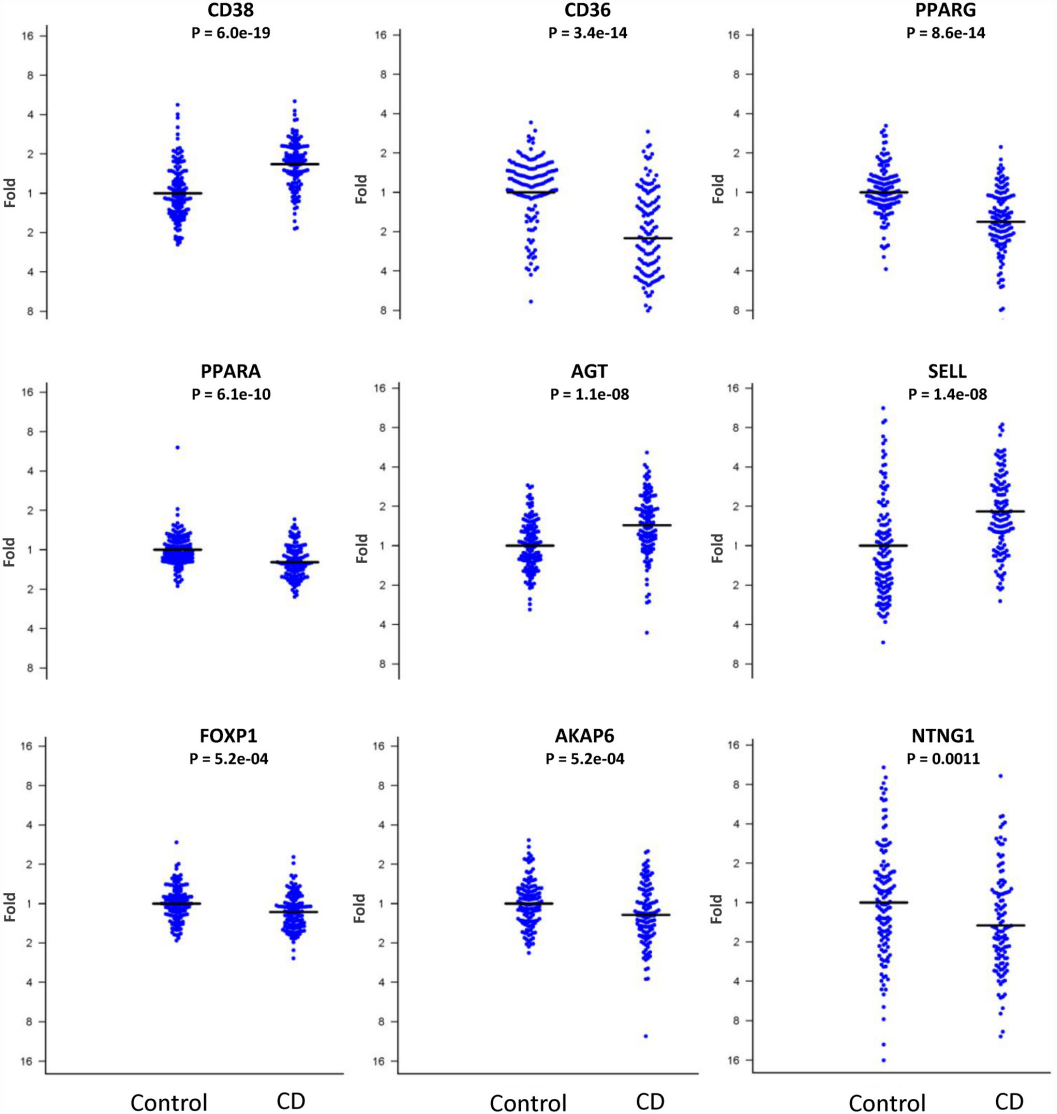
Nine target genes showing significant difference in gene expression between celiac disease (CD) patients and control patients. The y-axis shows the fold change gene expression and the mean expression in control patients is set to one.

Results from the 16 target genes from the main experiment and the fold change expression difference between the target genes and the reference “housekeeping” gene *IPO8* are presented in Table 1. Eleven genes reached a nominally significant p-value below 0.05, of which nine remained significant after adjusting for multiple comparisons using Bonferroni correction (Fig 1). Of those nine genes, *AKAP6* and *NTNG1* were genes associated with anorexia nervosa and the remaining seven (*AGT*, *CD36*, *CD38*, *FOXP1*, *PPARA*, *PPARG and SELL)* were selected for their relationship with T2D (S1 Table).

**Table 1:** Results from the main gene expression analysis. The Delta-Delta C_T_ (ΔΔC_T_) relative quantification method was used to estimate mRNA levels of target genes relative to a reference gene (*IPO8*) in the small intestinal biopsies from patients with celiac disease and compared with controls. A total of 144 cases and 154 control samples were analyzed. The p-value is calculated using the independent samples t-test for equality of means (equal variances assumed).

Gene	P-value	Mean Ct Difference	Std. Error	Lower	Upper	Bonferroni corrected p-value	FC	Percent change	Direction in CD vs Control
*CD38*	6.00E-19	0.74	0.08	0.59	0.89	2.40E-17	1.67	67%	UP
*CD36*	3.40E-14	1.19	0.15	-1.48	-0.9	1.36E-12	2.28	128%	DOWN
*PPARG*	8.60E-14	0.83	0.09	-1	-0.65	3.44E-12	1.77	77%	DOWN
*PPARA*	6.10E-10	0.33	0.05	-0.42	-0.23	2.44E-08	1.25	25%	DOWN
*AGT*	1.10E-08	0.59	0.09	0.42	0.76	4.40E-07	1.5	50%	UP
*SELL*	1.40E-08	0.88	0.15	0.59	1.17	5.60E-07	1.84	84%	UP
*FOXP1*	5.70E-05	0.22	0.05	-0.32	-0.12	2.28E-03	1.17	17%	DOWN
*AKAP6*	5.20E-04	0.29	0.08	-0.45	-0.12	0.02	1.22	22%	DOWN
*NTNG1*	1.10E-03	0.57	0.18	-0.92	-0.22	0.04	1.48	48%	DOWN
*BCL2L11*	1.70E-03	0.23	0.07	-0.38	-0.09	0.07	1.18	18%	DOWN
*PIEZO2*	6.70E-03	0.29	0.1	-0.49	-0.09	0.27	1.22	22%	DOWN
*FTO*	0.08	0.09	0.05	-0.01	0.19	1	1.07	7%	UP
*HFE*	0.09	0.19	0.11	-0.02	0.41	1	1.14	14%	UP
*ZNF804B*	0.11	0.43	0.28	-0.99	0.13	1	1.35	35%	DOWN
*ADRA1D*	0.18	0.36	0.22	-0.8	0.08	1	1.29	29%	DOWN
*KCNJ11*	0.53	0.1	0.12	-0.33	0.14	1	1.07	7%	DOWN

FC = Fold Change, CD = Celiac Disease

## Discussion

In this study we found that seven genes involved in T2D (*CD36*, *CD38*, *FOXP1*, *SELL*, *PPARA*, *PPARG*, *AGT) and t*wo genes involved in anorexia (*AKAP6*, *NTNG1)* were differently expressed in the small intestinal tissue of celiac patients compared with control patients, suggesting common genetic pathways leading to the disease among these phenotypically different chronic disorders. A plausible explanation could be that the genes discovered in this study are just an artifact of an inflamed tissue. However, except for *CD38*, *AGT and SELL*, the remaining genes are lower expressed in patients and therefore at least unlikely to be the result of an increased representation of immune cells in the intestinal tissue of celiac patients compared with controls.

The T2D related gene, *CD36*, is down regulated in celiac patients in this study. Studies on this gene showed its various functions in many processes including angiogenesis, inflammation, lipid metabolism, atherosclerosis, and platelet activation [19]. It has been shown that the *CD36* gene is linked with increased risk of T2D [20] and the CD36 protein level may serve as a biological marker of T2D [21]. Transgenic mice overexpressing *CD36* have reduced blood lipids and deficiency of *CD36* could lead to insulin resistance [22]. Our data show down regulation of *CD36* in celiac patients and the CD36 protein has also been previously shown to be significantly reduced in active as compared to inactive celiac disease and normal mucosal samples [23]. If this is a consequence of inflammation or a risk for developing both diseases needs to be addressed.

This study also showed that *CD38* was up-regulated in children with celiac disease. However, when the expression of this gene was normalized to *CD3D*, which is a part of the T-cell receptor, the difference was only nominally significant. The CD38 protein is like CD36, a surface molecule and *CD38* is expressed in human T and B cells during different stages of their development. A function of CD38 is mediating insulin secretion [24] and an immune response with auto-antibodies to CD38 protein is present in T2D patients [24, 25].

Another finding from this study was the down-regulation of mRNA levels of both *PPARA* and *PPARG* in celiac disease children as compared with controls. Peroxisome Proliferator-Activated Receptors (PPAR)-alpha and PPAR-gamma are two of three known subtypes of PPARs acting as transcription factors activated by ligands. Peroxisomes contain enzymes necessary for the oxidation process [26]. PPARs are known to regulate target genes of inflammatory responses as well as energy balance [27]. Importantly, PPARs have been found to act as anti-inflammatory and for these reasons PPARs have been considered for the development of new therapies for chronic inflammatory diseases [28]. Previous studies on *PPARG* revealed its contribution in numerous diseases, such as: obesity [29], T2D [30], and atherosclerosis [27] as well as in celiac disease [31]. It has also been shown that tTGA drives inflammation via *PPARG* down-regulation in celiac patients [31] and that down-regulation of proteins involved in PPAR signaling are associated with the highest celiac disease histological score [32].

Angiotensin-2, or the *AGT* gene was more expressed in the small intestinal mucosa of celiac disease children as compared with controls. *AGT* encodes a potent vasoconstrictor and acts directly on vascular smooth muscle and it is associated with T1D and T2D as well as with cardiovascular disease [33–35]. Interestingly, olmesartan, an angiotensin II receptor antagonist, has been shown to cause a severe form of sprue-like enteropathy [36]. *SELL* or L-selectin, encodes a cell surface adhesion protein which mediates the adherence of lymphocytes to endothelial cells. *SELL* is associated to both T2D [37] and amyotrophic lateral sclerosis (ALS) [38]. Both *AGT* and *SELL* are expressed at higher levels in celiac cases compared with controls indicating a high activity of adherence and interaction between leucocytes and endothelial cells.

The forkhead box P1 (*FOXP1*) is an essential transcriptional regulator for thymocyte development and the generation of quiescent naive T cells [39]. *BCL2L11* (which was slightly above the corrected p-value of 0.05) is important for apoptosis (cell death) and thymocytes lacking this pro-apoptotic Bcl-2 family member (also known as Bim) are refractory to apoptosis induced by TCR-CD3 stimulation [40]. *BCL2L11* has been identified as an essential initiator of apoptosis in thymocyte-negative selection of autoreactive T-cells [40]. *BCL2L11* and *FOXP1* seems to have an important role in normal thymocyte apoptosis and are both significantly down regulated in celiac cases compared with controls in this study.

*AKAP6* and *NTNG1* were previously reported in a GWAS of anorexia nervosa [41]. *AKAP6* is a member of A-kinase anchoring protein. Following the name, its main role is to attach enzymes and transport it near the target substrate. It is mostly expressed in brain and cardiac region as well as in skeletal muscles [42]. *NTNG1* (Netrin-G1) is a member of protein family playing an important role in the development of the human nervous system [43]. Previous studies revealed that mutations in *NTNG1* are associated with schizophrenia [44] and Parkinson’s disease [45].

In conclusion, even though the common mechanisms for the development of celiac disease, T2D or anorexia nervosa remains unresolved, the present study shows that several genes associated with T2D and anorexia nervosa are differentially expressed in children with active celiac disease as compared with controls, indicating a connection between these diseases.

## Materials and Methods

### Ethics Statement

This study has been conducted according to the principles expressed in the Declaration of Helsinki and approved by the Regional ethics board in Gothenburg. All guardians and study participants (when appropriate) gave their written informed consent. All personal data as well as results obtained from the research are coded and will remain confidential to all except for the treating physician at each hospital.

### Biological material

All children who were investigated at the Departments of Pediatrics with an upper endoscopy were consecutively asked to participate in the study as previously described [18, 46].

There were no exclusion criteria for participation. A child with a Marsh score >1 was considered to have CD and included as a case. A child with a Marsh score ≤1 was considered not to have CD and included as a disease control. In all, small intestinal biopsies and blood samples were collected from 144 cases with CD at a median age of 7.4 years (range 1.6–17.8) and from 154 disease controls at median 11.4.years (range 1.4–18.3). Among the disease controls, the most common diagnoses were gastroesophageal reflux disease and helicobacter pylori gastritis, whereas only eleven children had Crohn´s disease and four children ulcerative colitis, respectively. Another eight disease controls were detected with moderately elevated tTG antibody levels in a screening study and were considered to have potential CD.

### RNA extraction

Small intestinal biopsies were immediately put in a RNA stabilizing reagent, RNAlater solution (Life Technologies, CA, USA) and put in -4°C overnight in order to allow the reagent to penetrate the tissue. The biopsy was further frozen in -80°C until RNA extraction was carried out. Total RNA was extracted using the miRNeasy Mini Kit (QIAGEN, Germany) or the Maxwell^®^ 16 Total RNA Purification Kit (Promega) together with the Maxwell^®^16 instrument. The RNA quality and quantity was checked with a NanoDrop 2000 spectrophotometer and a 2100 Bioanalyzer (Agilent Technologies). RNA was converted to cDNA using the Vilo kit (Life Technologies, CA, USA).

### Reference gene validation

A total of 23 reference (housekeeping) genes were tested with the GeNorm algorithm [47]. The *IPO8* gene had the highest stability value (m<0.5) and was chosen as reference gene for normalization.

### Gene Expression Analysis

Quantitive gene expression analysis was performed by means of quantitative Polymerase Chain Reaction (qPCR) with TaqMan chemistry (Life Technologies, CA, USA). A total of 1 ng/reaction cDNA together with Master Mix was added to all genes simultaneously using a Nanodrop II dispenser (GC biotech, Netherlands), for a final reaction volume of 2 μl per gene and sample. QPCR reaction was run on the real-time PCR, ABI PRISM 7900HT Sequence Detection System (Life Technologies, CA, USA). Raw data was analyzed with the SDS 2.4 and RQ manager 1.2.1 software provided by the instrument.

### Gene selection criteria

The T2D risk genes were defined by the IPA analysis (Ingenuity Inc., CA, USA) in our previous GWAS [11]. All of these genes, except *PPARG*, were located in potential risk regions of celiac disease, with one or more nominally associated SNPs [11]. *PPARG* was chosen due to its relation to *PPARA*. We also included the nearest gene from four regions associated with anorexia nervosa [41] and overlapping with the results from our GWAS in celiac disease [11].

### Statistical analysis

Gene expression data was analyzed using the Delta-Delta C_T_ (ΔΔC_T_) relative quantification method[48]. This approach enables the expression of target genes to be normalized to reference genes, and then compared between cases and control samples (the ΔΔC_T_ value). To normalize the qPCR reaction for the amount of RNA added, the use of an internal reference gene (housekeeping gene) is used [48]. The threshold cycle (C_T_) or the cycle of quantification (Cq), is the PCR cycle when the amplification reaches a set threshold. Delta Ct (ΔC_T_) is calculated by the difference between the cycle of quantification for the target gene compared with the reference gene(-s). The t-test was used to calculate if there was a significant difference between the mean delta C_T_ value of the controls compared to the mean delta C_T_ of cases.

### URLs

Protein Database—UniProtKb/SwissProt: http://www.uniprot.org/

Entrez Cross Database: http://www.ncbi.nlm.nih.gov/sites/gquery

Gene Cards: http://www.genecards.org/

A Catalog of Published Genome-Wide Association Studies: www.genome.gov/gwastudies.

## Supporting Information

S1 File Phenotype information. This file gives information regarding CD diagnosis.
(TXT)

S2 File Ct values. This file gives the Ct value for each gene and patient.
(TXT)

S1 Table All tested genes including six genes previously run in Östensson et al. [11] and Montén et al. [18]. A list of all type 2 Diabetes and anorexia genes (including six genes were run as previously described. Forty-two genes were involved in type 2 diabetes and four were associated with anorexia. A total of forty target genes were analyzed in this study.
(DOCX)

## Abbreviations

GWAS: Genome-wide association studies
HLA: Human leukocyte antigen
PCR: Polymarease chain reaction
qPCR: Quantitative polymerase chain reaction
SNP: Single nucleotide polymorphism
T2D: Type 2 diabetes
T1D: Type 1 diabetes
tTGA: Tissue transglutaminase antibodies
TG2: Tissue transglutaminase
