# Supplementary material for: Shared Genetic Factors Involved in Celiac Disease, Type 2 Diabetes and Anorexia Nervosa Suggest Common Molecular Pathways for Chronic Diseases
Source: PLoS One. 2016 Aug 2;11(8):e0159593. doi: 10.1371/journal.pone.0159593 (PMC4970800; doi:10.1371/journal.pone.0159593)
Supplement: S1 Table — A list of all type 2 Diabetes and anorexia genes (including six genes were run as previously described. Forty-two genes were involved in type 2 diabetes and four were associated with anorexia. A total of forty target genes were analyzed in this study. (DOCX) [file pone.0159593.s003.docx]

**S1 Table. All tested genes including six genes previously run in Östensson et al. [11] and Montén et al. [18]** A list of all type 2 Diabetes and anorexia genes (including six genes were run as previously described. Forty-two genes were involved in type 2 diabetes and four were associated with anorexia. A total of forty target genes were analyzed in this study.

| Disease | Gene ID | Assay ID/info |  | Disease | Gene ID | Assay ID/info |  |
| --- | --- | --- | --- | --- | --- | --- | --- |
| T2D | *ABCC8* | Hs01093761_m1 |  | T2D | *PIEZO2* | Hs00401026_m1 |  |
| T2D | *ADRA1B* | Hs00171263_m1 |  | T2D | *PPARA* | Hs00947536_m1 |  |
| T2D | *ADRA1D* | Hs00169865_m1 |  | T2D | *PPARG* | Hs01115511_m1 |  |
| T2D | *AGT* | Hs01586213_m1 |  | T2D | *PRDM10* | Hs00999748_m1 |  |
| T2D | *AKAP10* | Hs00183673_m1 |  | T2D | *PRKCZ* | Hs00177051_m1 |  |
| T2D | *APOA5* | Hs00983449_g1 |  | T2D | *SELL* | Hs00174151_m1 |  |
| T2D | *ATP10A* | Hs00257114_m1 |  | T2D | *THBS2* | Hs01568063_m1 |  |
| T2D | *BCL2L11* | Hs00708019_s1 |  | T2D | *TXNIP* | Hs01006900_g1 |  |
| T2D | *BTC* | Hs01101204_m1 |  | T2D | *ZMYM2* | Hs00268802_m1 |  |
| T2D | *CACNA1A* | Hs01579431_m1 |  | Anorexia | *AKAP6* | Hs00188681_m1 |  |
| T2D | *CCR5* | Hs99999149_s1 |  | Anorexia | *CDH9* | Hs00940349_m1 |  |
| T2D | *CD36* | Hs01567185_m1 |  | Anorexia | *NTNG1* | Hs01552822_m1 |  |
| T2D | *CD38* | Hs01120071_m1 |  | Anorexia | *ZNF804B* | Hs00328367_m1 |  |
| T2D | *CMA1* | Hs00156558_m1 |  | T2D | *FABP1* | Östensson et al. NS |  |
| T2D | *CNTNAP2* | Hs01034283_m1 |  | T2D | *MTTP* | Östensson et al. NS |  |
| T2D | *CYBA* | Hs03044361_m1 |  | T2D | *RGS5* | Östensson et al. NS |  |
| T2D | *FOXP1* | Hs00212860_m1 |  | T2D/anorexia^a^ | *PPP3CA* | Montén et al. NS |  |
| T2D | *FTO* | Hs01057145_m1 |  | T2D | *INSR* | Montén et al. Sign |  |
| T2D | *HFE* | Hs00373474_m1 |  | T2D | *VEGFA* | Montén et al. NS |  |
| T2D | *HFE2* | Hs02378779_s1 |  | T2D | *INS* | not expressed |  |
| T2D | *KCNJ11* | Hs00265026_s1 |  | T2D | *TH* | not expressed |  |
| T2D | *KIRREL3* | Hs00419702_m1 |  | Reference | *CD3D* | Hs00174158_m1 |  |
| T2D | *KLF10* | Hs00921811_m1 |  | Reference | *GUSB* | Hs00939627_m1 |  |
| T2D | *MAPK1* | Hs01046830_m1 |  | Reference | *PGK1* | Hs99999906_m1 |  |
| T2D | *PBX3* | Hs00608415_m1 |  | Reference | *IPO8*^b^ | Hs00183533_m1 |  |

^a^ Associated with anorexia in Boraska V et al., February 11, 2014, Mol Psychiatry

^b^Reference gene used in the Delta-Delta C_T_ (ΔΔC_T_) relative quantification analysis.

NS = Not Significant, Sign = Significant

T2D = Type 2 Diabetes
